# Exploring Intra- and Inter-Regional Interactions in the IDP α-Synuclein Using smFRET and MD Simulations

**DOI:** 10.1021/acs.biomac.3c00404

**Published:** 2023-07-05

**Authors:** Gobert Heesink, Mirjam J. Marseille, Mohammad A. A. Fakhree, Mark D. Driver, Kirsten A. van Leijenhorst-Groener, Patrick R. Onck, Christian Blum, Mireille M.A.E. Claessens

**Affiliations:** † Nanobiophysics, Faculty of Science and Technology, MESA + Institute for Nanotechnology and Technical Medical Centre, University of Twente, PO Box 217, 7500 AE Enschede, The Netherlands; ‡ Micromechanics, Zernike Institute for Advanced Materials, University of Groningen, 9747 AG Groningen, The Netherlands

## Abstract

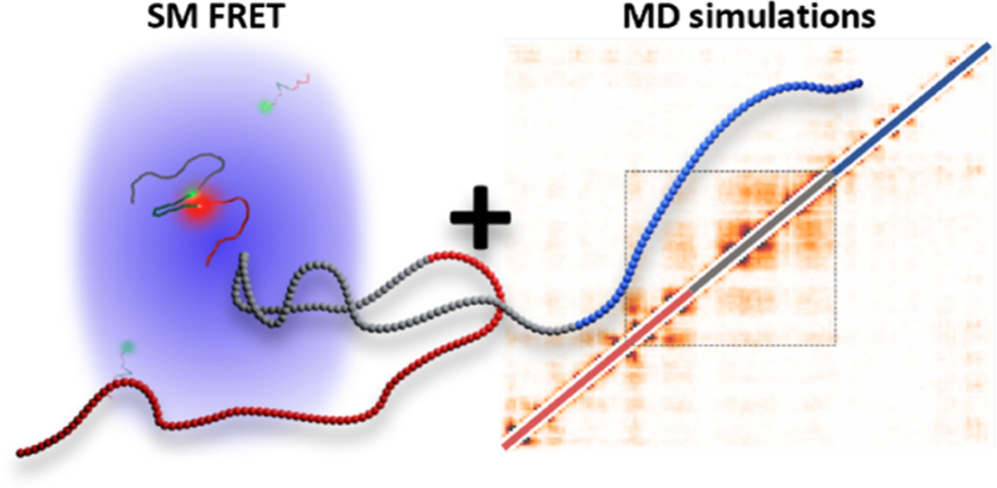

Theoretical concepts from polymer physics are often used to describe intrinsically disordered proteins (IDPs). However, amino acid interactions within and between regions of the protein can lead to deviations from typical polymer scaling behavior and even to short-lived secondary structures. To investigate the key interactions in the dynamic IDP α-synuclein (αS) at the amino acid level, we conducted single-molecule fluorescence resonance energy transfer (smFRET) experiments and coarse-grained molecular dynamics (CG-MD) simulations. We find excellent agreement between experiments and simulations. Our results show that a physiological salt solution is a good solvent for αS and that the protein is highly dynamic throughout its entire chain, with local intra- and inter-regional interactions leading to deviations from global scaling. Specifically, we observe expansion in the C-terminal region, compaction in the NAC region, and a slightly smaller distance between the C- and N-termini than expected. Our simulations indicate that the compaction in the NAC region results from hydrophobic aliphatic contacts, mostly between valine and alanine residues, and cation−π interactions between lysine and tyrosine. In addition, hydrogen bonds also seem to contribute to the compaction of the NAC region. The expansion of the C-terminal region is due to intraregional electrostatic repulsion and increased chain stiffness from several prolines. Overall, our study demonstrates the effectiveness of combining smFRET experiments with CG-MD simulations to investigate the key interactions in highly dynamic IDPs at the amino acid level.

## Introduction

α-synuclein (αS) is a 140 amino acid long intrinsically disordered protein (IDP)^1,2^ that is mainly known for its role in Parkinson’s disease, where it is assembled into amyloid fibril deposits known as Lewy bodies and Lewy neurites.^3^ Under physiological conditions, αS is abundantly expressed in neurons, where it most likely plays a role in membrane trafficking and remodeling processes.^4−8^ αS consists of three regions with distinctly different physicochemical properties. The N-terminal region from amino acids 1 to 60 is amphiphilic, the central NAC (non-amyloid-β component) region is more hydrophobic, and the C-terminal region from amino acids 95 to 140 contains many acidic amino acid residues. Despite being intrinsically disordered, αS adopts some degree of structure when it binds to other molecules. Upon binding to membranes, for instance, the N-terminal region of αS folds into an amphipathic helical structure.^9−11^ Other known αS interaction partners include the SNARE protein synaptobrevin, the cytoskeletal proteins actin and tau, and metal cations.^12−15^

The classification of αS as an IDP suggests that theoretical concepts from polymer physics can be used to describe its properties. Indeed, scaling laws that describe the dimensions of polymers have successfully been applied to IDPs and denatured proteins.^16^ Several studies report that αS can largely be described as a random coil polymer. NMR experiments hint at a slight disposition toward helical torsion angles in the N-terminal part of the protein in solution.^10^ trFRET studies on short αS segments have also shown subtle deviations from a fully disordered state in the N-terminal and NAC regions.^17^ Others reported that monomeric αS is present as an ensemble of more compact globular conformations with transient secondary structure elements.^18−20^ For a review of the structural features of monomeric αS, we refer to ref (21).

Establishing which molecular interactions are responsible for the temporal and spatial conformations of αS remains a challenge and has not been resolved today. Here, we combine single-molecule fluorescence resonance energy transfer (smFRET) studies with coarse-grained 1-bead-per-amino-acid (1BPA) molecular dynamics (CG-MD) simulations to gain insights into the conformation of αS at the amino acid level. smFRET studies have previously provided valuable information about the conformational dynamics of IDPs.^16,22−25^ smFRET experiments gave insights into the behavior of αS at different pH values,^26^ yet the quantification of distances for smFRET data remains a complex task. In our research, we investigate the dimensions and flexibility of full-length αS under physiological salt conditions. We combine intensity-based and lifetime-based smFRET data to accurately quantify distances. For the smFRET experiments, we produced 14 different FRET-labeled αS constructs, spanning the whole protein chain. We globally analyze the relationship between the measured distances (*R*) and the length of the αS segment between the labeling positions (Δ*N*) in the context of Flory theory for polymer chain configurations. In Flory theory, *R* ∼ Δ*N*^*ν*^, where the scaling exponent *ν* describes the solvent quality. The smFRET measurements allow us to observe local deviations from the global scaling behavior. We find an excellent agreement between the intramolecular distances measured in smFRET and the distances obtained from MD simulations. The agreement between experiments and MD simulations allows us to use the MD simulations to gain insight into the intra- and inter-regional contacts and the underlying key molecular interactions at the amino acid level.

We find that, along its full length, αS globally behaves as a polymer in a solvent with a Flory scaling exponent of *ν* = 0.57. This shows that physiological salt conditions resemble good solvent conditions for αS. Locally, we find compaction in the NAC region between amino acid positions 56 and 90, but surprisingly, the interactions in this region remain highly dynamic. In the C-terminal region, between amino acids 90 and 140, we find chain expansion. The CG-MD simulations show that the compaction in the NAC region is the result of hydrophobic contacts, especially between valine and alanine residues, and cation−π interactions between lysine and tyrosine. The expansion of the C-terminal part of the protein is a result of intrachain electrostatic repulsion and an increase in chain stiffness due to the presence of prolines. Additionally, we show that different physicochemical properties of the three protein regions also result in a different sensitivity to changes in solvent conditions.

## Materials and Methods

### FRET Labeling of αS Synuclein

A set of 14 αS variants, in which two amino acids were replaced with cysteines, were produced using standard biochemical tools.^27^ The following αS variants were created: αS_9–18_, αS_9–27_, αS_9–42_, αS_9–69_, αS_9–90_, αS_9–140_, αS_18–90_, αS_18–124_, αS_42–85_, αS_42–90_, αS_56–69_, αS_56–90_, αS_90–140_, and αS_130–140_. The substitutions to cysteines cover the different regions of the protein and vary in a number of amino acids between the two cysteines. FRET labeling of the cysteines was done using a maleimide–thiol reaction following the protocol described previously.^11,28^ Alexa Fluor 488 was used as the FRET donor dye and Alexa Fluor 568 as the FRET acceptor dye. The FRET-labeled samples were aliquoted, stored at −80 °C, and freshly thawed before the experiments.

### smFRET Instrumentation, Data Collection, and Analysis

The smFRET burst traces were recorded using an ultrasensitive custom-built confocal microscope that was described in detail elsewhere.^11,29^ In short, a pulsed diode laser operating at 485 nm (PDL800-D, PicoQuant, Germany) was used as the excitation source. A Plan Apo VC, 60×, 1.2NA, Nikon microscope objective was used to excite and collect the emission from the samples. A 585 nm dichroic beam splitter (T585lpxr, Chroma) separated the emitted light into two channels: a green, FRET donor channel for detection at wavelengths shorter than 561 nm (RazorEdge, 561 nm short pass, Semrock) and a red, FRET acceptor channel for detection from 590 to 770 nm (590 nm long pass, Olympus, Japan, in combination with a BrightLine 770 nm short pass, Semrock). For each channel, a 15 μm pinhole was used to filter the emission light spatially. The light was subsequently focused onto single-photon avalanche diodes (Excelitas SPCM-AQR-56) connected to a TCSPC module (PicoHarp300, PicoQuant, Germany) to determine the arrival time of all individual photons relative to the respective excitation pulse.

For the experiments, the FRET-labeled αS variants were diluted in phosphate-buffered saline (137 mM NaCl, 2.7 mM KCl, 10 mM Na_2_HPO_4_, 1.8 mM KH_2_PO_4_, pH 7.4, 1× PBS), saturated with Trolox to quench the triplet state and to suppress the dark states of the fluorophores.^30^ To investigate the effect of solvent quality on the αS conformation, experiments were performed in a low-salt buffer (0.1× PBS), 5 M urea, and 40% methanol. To obtain fluorescence time traces, samples were diluted to concentrations of approximately 100 pM, resulting in 1 to 10 bursts per second on average. The diluted FRET-labeled αS samples were deposited on microscope cover glasses. Prior to sample deposition, the cover glass surface was saturated with nonlabeled αS to reduce unspecific binding of the FRET-labeled αS to the glass surface. All smFRET measurements were performed in solution, approximately 10 μm above the coverslip surface. For each αS variant, at least two 30 min long fluorescence time traces were recorded.

Recording the fluorescence over time gives a dataset containing the arrival time of each detected photon, recorded with respect to both the start of the experiment (macro time) and the excitation pulse (micro time). Photon macro times are binned using a bin time of 1 ms to obtain a burst trace, showing the bursts of emission from the FRET donor and acceptor over time. The intensity was corrected considering the background fluorescence, leakage of the donor signal into the acceptor channel, differences in fluorescence quantum efficiencies of the FRET donor and acceptor dyes, and differences in the detection efficiencies of the two detection channels as outlined by Hellenkamp.^31^ Cross excitation of Alexa Fluor 565 by 485 nm excitation was neglected. Corrections for the differences in efficiencies were determined globally for the setup and specific dye pair.

The corrected intensity burst trace was analyzed using a minimum threshold of 50 photons for the sum of donor and FRET acceptor emission to discriminate between the signal and noise. To filter out signals from the aggregated protein, we applied a maximum threshold of 1000 photons and neglected the signal from slowly diffusing species that are present in the detection volume for more than 1 ms.

For the analysis, we separated bursts containing only the donor signal (donor-only bursts) from bursts containing the signal from both the FRET donor and acceptor (FRET bursts). For each FRET burst, we determined the FRET efficiency (*E*_FRET_^burst^) based on the acceptor emission intensity (*I*_A_^burst^) and the donor emission intensity (*I*_D_^burst^) as *E*_FRET_^burst^ = *I*_A_^burst^/(*I*_A_^burst^ + *I*_D_^burst^).

To obtain the burst-integrated average photon arrival time per burst, relative to the excitation pulse, we used the instrument response function (IRF) to correct for the time offset. To test the validity of the correction, we determine the average fluorescence photon arrival time of freely diffusing Alexa Fluor 488 in single-molecule experiments. The obtained average arrival time of 4.1 ns agrees well the with expectations. To analyze the data obtained for the FRET-labeled αS variants, we determined for each donor-only burst a τ_D_^burst^ and for each FRET burst a τ_DA_^burst^, respectively. For each of the FRET-labeled αS constructs, we determined the average τ_D_ from the highest density point in the τ_D_^burst^ versus *E*_FRET_^burst^ data, using Gaussian kernel probability density estimation.

The smFRET data obtained for the FRET bursts was subsequently plotted as τ_DA_^burst^/τ_D_ versus *E*_FRET_. In these plots, each data point represents one burst. From these plots, we determined the τ_DA_ analogue to how τ_D_ was determined. All analyses were performed in Matlab 2020b.

### Modeling E_FRET_ and τ_DA_/τ_D_ as a Function of the Mean End-to-End Distance R

*E*_FRET_ and equivalently (1 – τ_DA_/τ_D_) directly relate to the root-mean-square end-to-end distance of the regions of the protein between the FRET labels, ⟨*r*^2^⟩^1/2^ = *R*. The mean FRET efficiency is given by *E*_FRET_ = ∫*E*(*r*) × *P*(*r*) d*r*, in which *r* describes the distance between the donor and acceptor dyes, *E*(*r*) describes the distance dependence of the energy transfer process, and *P*(*r*) describes the probability density function of distances; in modeling *E*_FRET_ and τ_DA_/τ_D_, we assume that for αS, *P*(*r*) can be described as self-avoiding random coil. We used a general form of *P*(*r*) that takes into account the Flory scaling exponent *ν* as defined by^32,33^
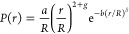
1where *g* = (γ – 1)/*v*, with γ = 1.1615^32^ and δ = 1/(1 – *v*). The normalizing constants *a* and *b* are calculated using ∫*P*(*r*) d*r* = 1 and ∫*r*^2^*P*(*r*) d*r* = *R*^2^. We used *R* = 5.5 nm to determine *a* and *b* for *ν* = 0.3, 0.4, 0.5, 0.6, and 0.7. Spline and linear interpolation were used to obtain values for *a* and *b* as a function of *ν*, respectively (Figure S2). Changes in *a* and *b* with changes in *R* are negligible in the range of interest and are hence ignored.

The mean relative donor lifetime τ_DA_/τ_D_ depends on the distribution of donor photon arrival times. This distribution follows from the weighting that *P*(*r*) imposes on the donor photon arrival times, as a result of the inverse relation between the energy transfer rate and the probability of emitting a donor photon. Given that the dynamics of the protein is slower than the donor lifetime, the effect of *P*(*r*) on τ_DA_/τ_D_ can be described as^34^
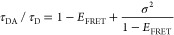
2where σ^2^ refers to the FRET efficiency variance, defined as
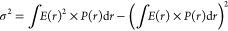
3Finally, the measured *E*_FRET_ and τ_DA_/τ_D_ are converted into *R*_int_ and *R*_τ_. The relation between the measured *E*_FRET_ and τ_DA_/τ_D_ depends on the Flory scaling exponent *ν*. Because the *E*_FRET_ and τ_DA_/τ_D_ values both report on the donor–acceptor distance, the values of *R*_int_ and *R*_τ_ should be identical if the correct value of the Flory scaling exponent *ν* is used. Hence, minimizing the difference between *R*_int_ and *R*_τ_ as a function of *ν* results in *ν* that globally best describes αS’s behavior.

The resulting values of *R*_int_ and *R*_τ_ are plotted versus the amino acid difference (Δ*N* = *N*_j_ – *N*_i_) for all αS constructs. Using the obtained optimal value found for *ν*, we fit the R-data to the Flory model for polymer chain configurations (eq 4), based on nonlinear least squares fitting using the Trust-Region algorithm, to obtain the persistence length *l*_p_ of the protein. In this fit, we assume a bond length of 0.38 nm and consider only values of *R* for which *E*_FRET_ < 0.9. The contribution of the dye and linker to values of *R* was accounted for by an additional 4 amino acids. All modeling were performed in Matlab 2020b.

## MD Simulations

CG-MD simulations were run using an updated 1-bead-per-amino-acid (1BPA) model^35−37^ with GROMACS 2019.6^38^ using implicit-solvent Langevin dynamics with an inverse friction coefficient of 50 ps and a timestep of 0.02 ps. The bond length between neighboring amino acids is 0.38 nm and an average mass of 120 kDa is assigned to each amino acid bead. Production runs were executed for 3 μs, with the first 0.5 μs discarded as equilibration. The 1BPA model contains sequence-specific backbone bending and torsion potentials described in ref (36, 37). The nonbonded interactions consist of a modified Coulomb law for electrostatic interactions, including Debye screening and solvent polarity, a shifted Lennard-Jones 8–6 potential for hydrophobic/hydrophilic interactions,^35−37^ and cation−π interactions by a Lennard-Jones 8–6 potential. We used the 1BPA 2.0 updated nonbonded parameters developed by Driver and Onck in this work. Simulations were performed on the αS sequence and the sequences with the cysteine mutations used for FRET labeling (no fluorophores attached). The simulation of the protein conformations was processed, and the mean (end-to-end) distance between the labeled residue pairs was computed.

To account for the presence of the dyes in the simulations, we approximate the dyes as noninteracting rigid beads, such that the distance between the *C*_α_ of the attachment site and the center of the dye is approximately twice the bond length between two amino acids, yielding a distance of 0.76 nm per dye. The dye residues are free to rotate about the protein chain, such that they will not always be maximally separated. Using the assumptions that the dyes are in a good solvent, and behave like a self-avoiding random coil, the extra distance becomes 0.76 × 2^3/5^ = 1.15 nm for the 2 dye–peptide bonds.^39^

Computation of intramolecular contacts was done using a cutoff distance of 0.7 nm between residues. Intramolecular contacts were ignored between residues with a difference in residue index of 3 or less. The simulation data was processed using MDAnalysis.^40,41^

## Results and Discussion

**Figure 1:**
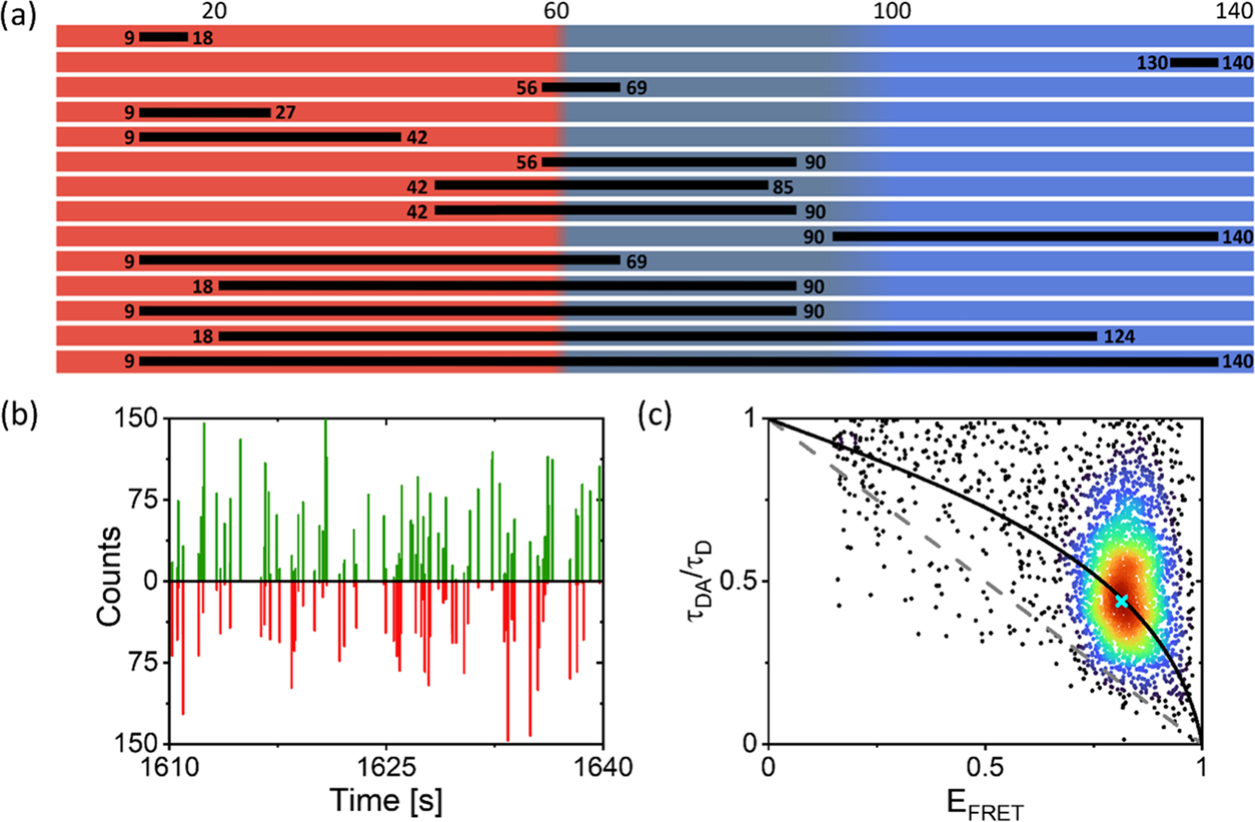
(a) Overview showing the 14 αS constructs used to probe intramolecular distances in the full-length αS. The labeling positions are indicated by the amino acid numbers connected by a black line. The N-terminal region is indicated in red, the NAC region in gray, and the C-terminal region in blue. (b) Zoom-in of an smFRET burst trace recorded for the FRET-labeled αS_9–42_ construct. Upon FRET donor excitation, emission in the FRET donor channel (green) and the acceptor channel (red) was recorded in time. The individual burst originates from single FRET-labeled αS proteins that diffuse through the detection volume. (c) Relative donor fluorescence lifetime τ_DA_^burst^/τ_D_ versus *E*_FRET_^burst^ of the FRET-labeled αS_9–42_ construct. Each point in the plot represents one FRET burst. The color scale from dark blue to dark red represents the density of data points from low to high density, respectively. The highest density center of the data is indicated with a cyan cross. The dashed gray line represents the static line (absence of conformational dynamics). The black line represents the dynamic line and was calculated using the distance distribution expected for a self-avoiding polymer chain in a good solvent.

To detect the intra- and inter-regional interactions present in αS, we use 14 different FRET-labeled αS constructs (Figure 1a). For all FRET-labeled αS constructs, we recorded single-molecule burst traces. A typical burst trace for the αS_9–42_ construct is shown in Figure 1b. In the traces, the presence of single proteins that diffuse through the detection volume is clearly visible as well-separated emission bursts. Some of the FRET donor fluorophore bursts (green) do not coincide with a FRET acceptor fluorophore burst (red). These donor-only bursts originate from proteins that only contain (1 or 2) donor fluorophores and are used to determine the donor-only lifetime τ_D_. Since these bursts do not contain any FRET information, they are not considered in the further analysis. For each FRET burst, the average FRET efficiency was obtained from the ratio of the emission intensities of the donor and acceptor fluorophore. We refer to this burst-integrated FRET efficiency as *E*_FRET_^burst^. For each protein, *E*_FRET_^burst^ reflects the average distance between the FRET labels while the protein was diffusing through the detection volume. Additionally, we determined the burst-integrated average donor photon arrival time (τ_DA_^burst^) from the recorded data and normalized τ_DA_^burst^ to the average fluorescence lifetime of the FRET donor in the absence of a FRET acceptor (τ_D_). τ_DA_^burst^ relates to the conformational dynamics of the protein on sub-millisecond time scales. Typically, smFRET data is presented as τ_DA_^burst^/τ_D_ versus *E*_FRET_^burst^.^34,42−44^ In Figure 1c, the τ_DA_^burst^/τ_D_ versus *E*_FRET_^burst^ data is plotted for the αS_9–42_ construct. Each data point represents a time bin of 1 ms and thus reflects the average conformation of an αS protein during the passage through the detection volume. From the data, we determine the highest density center of the data cloud to obtain the average τ_DA_/τ_D_ and E_FRET_ for the αS_9–42_ construct.

Generally, τ_DA_/τ_D_ is expected to linearly decrease with *E*_FRET_ when the distance between the FRET pairs does not change while the protein diffuses through the detection volume. This linear relation between τ_DA_/τ_D_ and *E*_FRET_, however, only holds if the labeled molecule’s conformation does not change while it diffuses through the detection volume. This linear relation is shown as the gray dashed line in Figure 1c and we refer to this relation as the static line. For FRET-labeled proteins that are dynamic and undergo conformational fluctuations while diffusing through the detection volume, τ_DA_/τ_D_ versus *E*_FRET_ lies above the static line (τ_DA_/τ_D_ – *E*_FRET_ > 0). The protein dynamics leads to deviations from the mean distances to shorter and longer distances. These deviations to shorter and longer distances contribute differently to the FRET process and hence affect the observed average donor photon arrival time. This leads to τ_DA_/τ_D_ – *E*_FRET_ > 0 for molecules that undergo conformational fluctuations on time scales that are much shorter than the passage time through the detection volume. For αS, the global chain reconfiguration occurs on time scales of approximately 60 ns, much faster than the passage time of some hundreds of microseconds.^45^ Taking into account the distance distribution corresponding to a polymer in a good solvent (*ν* = 3/5), the resulting probability density function of donor photon arrival times allows us to model the relation between *E*_FRET_ and τ_DA_/τ_D_. The result is shown in Figure 1c as a solid black line, which is referred to as the dynamic line. For the αS_9–42_ construct, the single burst data points lie above the static line and around the dynamic line (Figure 1c). The center of the data cloud, indicated with a cross, lies on the dynamic line demonstrating the intrinsically disordered nature of the region of the αS protein in between amino acid positions 9 and 42. Under physiological salt conditions, the region between amino acid positions 9 and 42 can be described well as a polymer in a good solvent.

**Figure 2:**
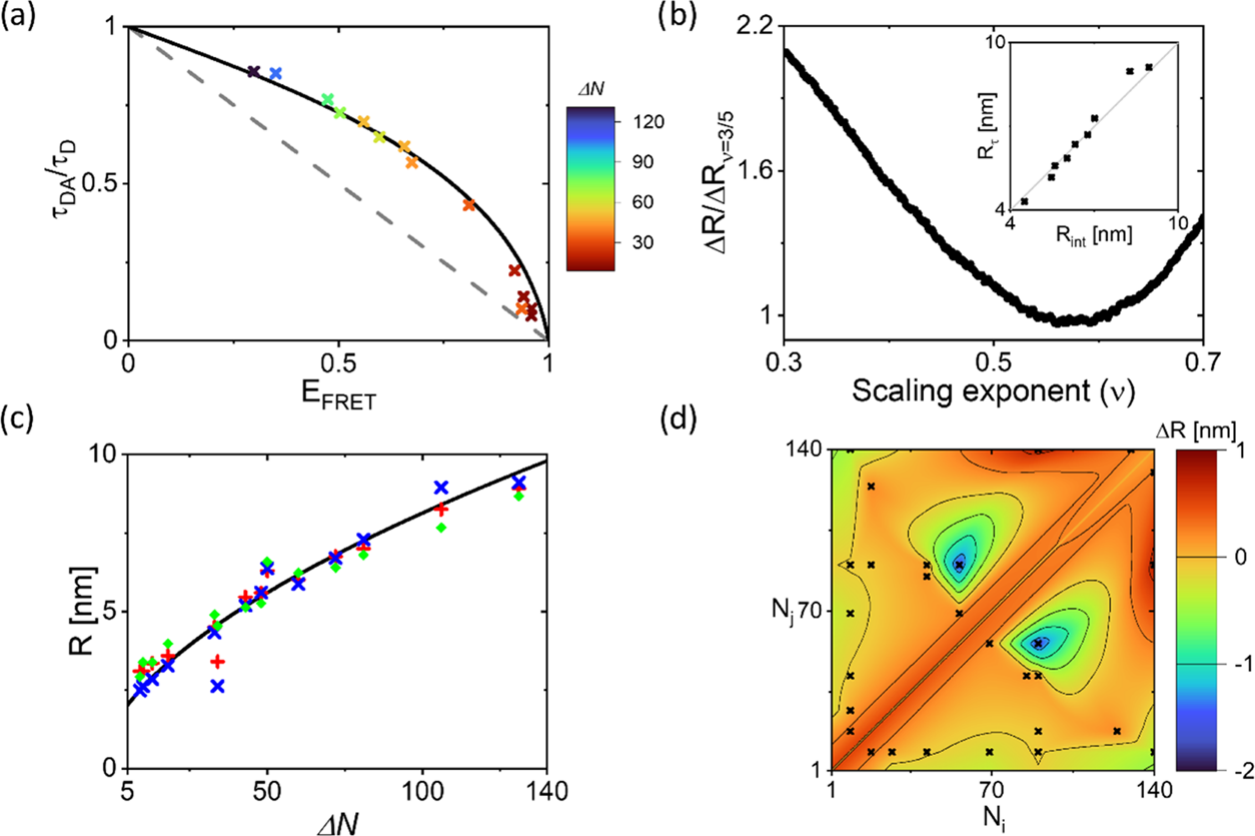
(a) Highest density points obtained from the τ_DA_^burst^/τ_D_, *E*_FRET_^burst^ data of all 14 FRET-labeled αS constructs. The number of amino acids between the FRET labeling positions (Δ*N*) is color coded as indicated by the color bar. The static line (dashed, gray) and the dynamic line (black) are also shown. (b) Calculated average difference between *R*_int_ and *R*_τ_ relative to good solvent conditions Δ*R*/Δ*R*_*ν*=3/5_ as a function of the Flory scaling exponent *ν*. *R*_int_ and *R*_τ_ are obtained for each construct obeying *E*_FRET_ < 0.9, using the center *E*_FRET_ and τ_DA_ data, respectively. Δ*R*_normalized_(*ν*) has a minimum at *ν* = 0.57. The inset shows *R*_int_ vs *R*_τ_ obtained for *ν* = 0.57 (normalization constants: *a* = 4.006 and *b* = 1.352). (c) The root-mean-square end-to-end distance *R* calculated from the *E*_FRET_ (red) and τ_DA_/τ_D_ (blue) data, taking into account the distance distribution and a Flory scaling exponent of 0.57, plotted as a function of the number of amino acids between labeling positions (Δ*N*). To obtain *l*_p_, the data was fitted to eq 4 using a Flory scaling exponent of 0.57 and a bond length of 0.38 nm (black line). The dye–linker distance was taken into account. From this fit, we obtain *l*_p_ = 0.49 nm. MD simulations of the αS chain conformations result in the distances shown in green. (d) Deviation in the average distance from the global fit using *ν* = 0.57 observed for the different labeling positions as determined from both *E*_FRET_ and τ_DA_/τ_D_ data. The black crosses represent the data points. The data is interpolated using natural neighbor interpolation and this interpolation is shown in color. A negative deviation is observed for the amino acids between positions 56 and 90 showing chain contraction. Between amino acid positions 90 and 140, we find a positive deviation, and the chain is slightly more extended. The distance between amino acid positions 9 and 140 is also slightly smaller compared to the global fit.

To investigate if the other regions of the protein behave similarly, the smFRET experiments were repeated for the other 13 FRET-labeled protein constructs. For each of these constructs, we obtained *E*_FRET_ and τ_DA_/τ_D_ from the density centers of the single burst data clouds (Figure S1). The center *E*_FRET_ and τ_DA_/τ_D_ values are plotted in Figure 2a for all 14 αS constructs. We observe that for most points, the center *E*_FRET_ values increase and center τ_DA_/τ_D_ values decrease with an increasing number of amino acids between the labeling positions. All of the 14 points lie above the static line close to or on the dynamic line. This evidences the existence of sub-millisecond conformational dynamics in all parts of the αS protein. Overall, the data points are close to the calculated dynamic line. This first observation suggests that physiological salt conditions are close to a good solvent for the protein. To assess if local deviations from the global behavior exist and to be able to quantify these deviations, we first determine the scaling exponent that globally describes the behavior of αS in physiological salt conditions best.

To do this, we convert the observed values for *E*_FRET_ and τ_DA_/τ_D_ into root-mean-square end-to-end distances. For each data point, this results in two calculated root-mean-square end-to-end distances, one based on the intensities of the FRET donor and acceptor fluorophores *R*_int_ and one based on the donor average photon arrival time *R*_τ_. To calculate *R*_int_ and *R*_τ_, the probability density function of the end-to-end distance has to be accounted for. This probability density function depends on the assumed solvent quality and hence on *ν* (see SI, Figure S3). For *R*_τ_, the probability density function of the photon arrival times additionally has to be taken into account. As a result, *R*_int_ and *R*_τ_ depend differently on *ν*. We use this difference to obtain the *ν* that globally best matches the data derived from the measurements. We vary *ν* to minimize the difference between *R*_int_ and *R*_τ_. Figure 2b shows the difference in Δ*R*/Δ*R*_*ν*=3/5_ obtained for the different values of *ν*. In the minimization, we take into account all data points for which *E*_FRET_ < 0.9. For *E*_FRET_ > 0.9, the number of detected donor photons is inherently low, which limits the accuracy in *E*_FRET_. The best agreement is observed for *ν* = 0.57, which is indeed close to the good solvent conditions used to obtain the dynamic line (Figures 1c and 2a). In the inset of Figure 2b, we show the *R*_int_ and *R*_τ_ assuming *ν* = 0.57.

In Figure 2c, the obtained values for *R*_int_ and *R*_τ_ are plotted as a function of the number of amino acids between the labeling positions, Δ*N*. We globally fit this dataset to the Flory model for polymer chain configurations^16^ using *ν* = 0.57
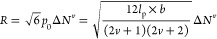
4In this expression, *l*_p_ is the persistence length and *b* is the bond length of the polymer chain. We obtain a global value for the persistence length of the αS chain, *l*_p_ = 0.49 nm. This value for *l*_p_ agrees well with earlier findings.^46^ As expected, the fitted curve globally describes the data points. However, some deviations can be observed. To visualize for which regions of αS, we experimentally find deviations from the global scaling behavior, we plot Δ*R*, the difference between the averaged *R*_int_ and *R*_τ_ data and the distance assuming the global scaling behavior, in Figure 2d. For the region between amino acids 90 and 140, we find a positive Δ*R*, which shows that the C-terminal region is extended. For the central region of the protein between amino acids 56 and 90, we find a negative Δ*R*, so this region is more compact. The distance between both ends of the protein between amino acids 9 and 140 is slightly smaller than expected for *ν* = 0.57.

**Figure 3:**
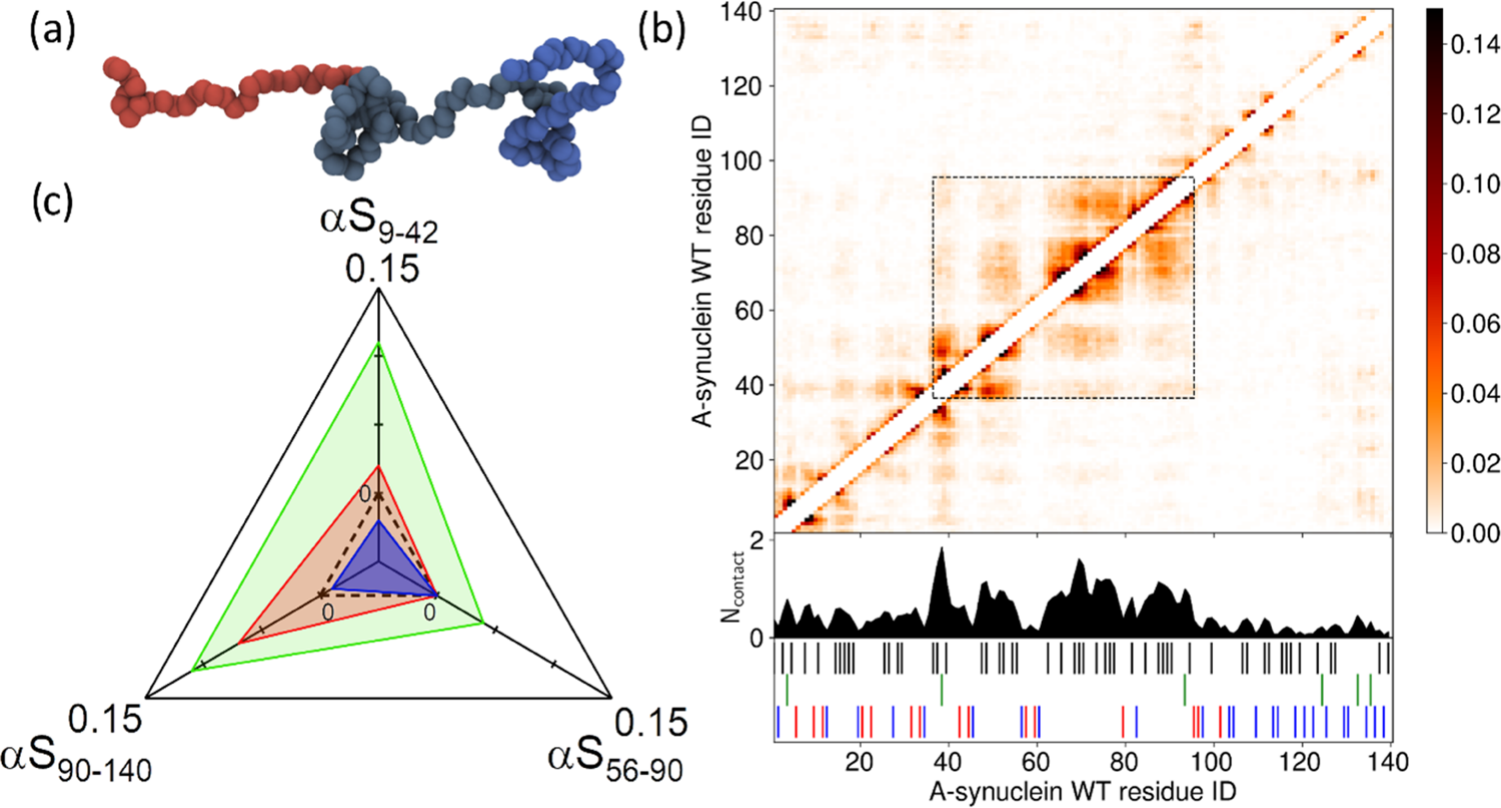
(a) Snapshot from the 1BPA CG-MD simulation of αS. The N-terminal region is indicated in red, the NAC region in gray, and the C-terminal region in blue, corresponding to the colors used in Figure 1a. A trajectory movie can be found in the Supporting Information. The protein has an average asphericity of 0.38 (±0.19) and a shape parameter of 0.45 (±0.40), indicating a preference for cylindrical conformations. (b) Time-averaged contact map for αS by amino acid number. The intensity scaling refers to the average amount of time (as a fraction of the total simulation time) two amino acids are in contact. The dashed box highlights that most of the interactions occur within the central region of the protein, including the NAC region and part of the N-terminal region. The one-dimensional summation of the interactions is shown below the map. *N*_contact_ refers to the average number of contacts an amino acid has at any given time. The amino acids are categorized into five groups: cationic (R, K)—red, anionic (D, E)—blue, aromatic (F, Y, W)—green, hydrophobic aliphatic (A, C, I, L, M, P, V)—black, and hydrophilic (G, N, S, H, Q, T)—white. (c) Observed difference between the *E*_FRET_ measured in physiological salt conditions and the *E*_FRET_ measured in low salt (red), in 5 M urea (green), and in 40% methanol (blue). Data is shown for the three different protein regions. The N-terminal region is represented by the FRET-labeled αS_9–42_ construct, the NAC region by the αS_56–90_ construct, and the C-terminal region by the αS_90–140_ construct. The dashed black triangle represents no change (Δ*E*_FRET_ = 0). In low salt, we observe chain expansion in the C-terminal and N-terminal regions. In 5 M urea, all regions expand. In 40% methanol, we observe a minor compaction of the N-terminal region.

To further investigate the nature of these deviations from the global scaling, we performed CG-MD simulations based on a 1-bead-per-amino-acid (1BPA) model that was developed for IDPs.^35−37^ The model discriminates between all 20 amino acids and accounts for a residue-specific backbone stiffness in addition to nonbonded hydrophobic, cation−π, and electrostatic interactions. A snapshot from the simulations is shown in Figure 3a, and a movie depicting a full simulation can be found in the Supporting Material. From these simulations, the mean distance between the labeling positions for each αS construct was obtained. The average distances *R* are shown in Figure 2c as green data points, showing an excellent agreement with the smFRET data. This agreement is rewarding given the fact that our CG-MD simulations are pure predictions; no parameter has been adjusted or fitted to make the simulations match the experimental data.

To better understand where in the protein sequence the deviations from the overall chain behavior occur, a time-averaged intramolecular contact map was created (Figure 3b). Amino acid pairs are assumed to be in contact if they are relatively positioned within 0.7 nm.^47^ In the one-dimensional summation of the contacts, the difference in occurrence of intramolecular contacts between the three different regions of the protein can be clearly observed. Intramolecular contacts are most abundant in the central region of the protein that includes the NAC domain and part of the N-terminal domain. This domain is enriched in hydrophobic aliphatic amino acid residues. The higher probability of intramolecular contacts indicates a relative compaction of this region. This compaction mainly results from hydrophobic self- and cross-interactions of valine and alanine residues (Figure S4a). Intramolecular contacts are least abundant in the C-terminal region of the protein. This region is rich in prolines, increasing the persistence length and thus stiffening the chain.^36,48^ Further, this region contains many acidic, negatively charged, amino acid residues, which leads to intrachain repulsion. Both factors contribute to the more extended conformations in the C-terminal region of the protein. The contact map also shows an increased probability of contacts between the C-terminal region (amino acids 125–135) and amino acids in the N-terminal region and between the C-terminal region (amino acids 125–135) and the C-terminal region around amino acid 100. The increased probability of contacts can be attributed to cation−π interactions between lysine and tyrosine (Figure 3b). These interactions are even better visible in time-averaged contact maps by residue type normalized to amino acid abundance (Figure S4b); other electrostatically mediated interactions between the C-terminal and N-terminal domains seem to not play a role. Cation−π interactions between tyrosine in the C-terminal and lysine in the N-terminal region explain the slightly decreased average distance observed in the smFRET experiment for the αS_9–140_ construct compared to the global fit, even though the C-terminal region itself is more extended (Figure 2c).

It should be noted that all-atom MD simulations can be performed in the full-length αS. However, the force fields have been parametrized with respect to folded proteins and do therefore have the tendency to be overly sticky. This results in an underprediction of the radius of gyration for αS of ∼2 nm,^20,49^ compared to experiments (3.3 ± 0.3 nm) and our CG-MD simulations (3.3 ± 0.8 nm).^19^ In addition, the end-to-end distance of full-length αS can be estimated from Figure 2c to be between 9 and 10 nm for the smFRET and CG-MD data, which is considerably larger than ∼5 nm observed for all-atom MD simulations.^49^ We also note, however, that our model does have limitations. This becomes apparent in the deviation between experimental and computational results for the measurements between residues 56 and 90 in the αS_56–90_ construct. All-atom MD simulations show the presence of hydrogen bonds and a significant probability for secondary structure formation (β strands) in this region. This leads to a level of compaction that our CG-MD simulation is unable to reproduce.^20,49^ For all other smFRET measurements, the very good agreement between the CG-MD simulations and measurements indicates that we accurately capture the dynamic properties of the protein.

The finding that some regions in αS are more compact or extended compared to the global scaling behavior of the protein chain shows that physiological salt conditions are not a good solvent for the entire chain. Changes in solvent conditions should therefore affect the protein regions differently. We test this by changing the solvent to (1) a low-salt buffer, which will affect intrachain repulsion by charge screening, (2) a 5 M urea solution, which is commonly used to denature proteins by solubilization and should affect the compaction due to hydrophobic interactions, and (3) a 40% methanol solution, which is commonly used to decrease solvent quality for proteins. We performed smFRET measurements in these solvents for three different αS constructs (αS_9–42_, αS_42–90_, and αS_90–140_), which cover the amphiphilic, NAC, and acidic region of αS, respectively. For all regions in all solvents, the τ_DA_/τ_D_ values of the freely diffusing proteins shift compared to the values obtained in physiological salt conditions. Our data showed that the protein remains flexible in these solvents (τ_DA_/τ_D_ – *E*_FRET_ > 0). Because the solvent conditions changed, we could not use the parameters obtained at physiological salt conditions to determine average distances *R*. Instead, we directly show the difference between the *E*_FRET_ values obtained in the physiological salt conditions and those in the modified solvents (Δ*E*_FRET_). Positive values of Δ*E*_FRET_ represent chain expansion, and negative values of Δ*E*_FRET_ represent compaction. The obtained Δ*E*_FRET_ for freely diffusing αS in low salt, urea, and methanol is shown in Figure 3b.

In the low-salt buffer, we observed no significant Δ*E*_FRET_ for the 9–42 and 42–90 regions (Figure 3c). For the 90–140 region, Δ*E*_FRET_ is positive, indicating that this region expands in the low-salt buffer. This matches expectations that the Debye length is increased resulting in long-range electrostatic repulsion. This confirms that intramolecular electrostatic repulsion contributes to the relative expansion of the 90–140 region in physiological salt conditions.

In 5 M urea, we observe an increase in Δ*E*_FRET_ for all three protein regions tested (Figure 3c). Although we do not observe any signature of the secondary structure of αS in physiological salt conditions, urea is able to further expand all tested regions of this IDP. Urea preferentially binds to all three regions of the protein since the dispersion interactions between urea and the protein backbone and side chains are stronger than for water.^50^ Additionally, hydrogen bonds are formed between urea and the carbonyl and amide groups in the backbone.^50^ Combined, these interactions with urea are probably responsible for the substantial increase of the stiffness and extension observed for unstructured peptides and likely result in the observed increase in Δ*E*_FRET_ for the three αS regions tested here.^51^

The addition of methanol does not result in the expected compaction of the αS monomers. Instead, we observe a large population of slowly diffusing particles upon the addition of methanol. This implies that the presence of methanol indeed decreases the solvent quality, which leads to the aggregation rather than compaction of most of the monomeric αS, even at the low picomolar protein concentrations used in the smFRET experiments. The effect of methanol on oligomerization and aggregation has been reported before but for much higher αS concentrations.^52^ For the analysis of Δ*E*_FRET_, we only consider the small fraction of the particles diffusing through the confocal volume for which the diffusion time remains low, indicating that these are protein monomers. Surprisingly, for these single freely diffusing individual proteins, the decrease in solvent quality by the addition of methanol does not result in a significant negative Δ*E*_FRET_, we only observe a minor compaction in the N-terminal region of the protein (Figure 3c). The limited amount of data does not give insights into whether compaction did not occur in the rest of the protein or if compaction is balanced by, e.g., the appearance of the local structure.

## Conclusions

We find that along the whole chain, the protein αS is highly dynamic. The root-mean-squared distances between the FRET labeling positions as a function of the segment lengths in the number of amino acids follow Flory theory with a scaling exponent of 0.57. At physiological salt conditions, the soltuion is globally a good solvent for the IDP αS. However, locally, we observe intra- and inter-regional interactions that result in deviations from the global scaling. Although all regions are highly dynamic, the C-terminal region of αS is somewhat expanded while we observe compaction in the NAC region. In addition, the distance between the C-terminus and N-terminus of the protein is slightly smaller than expected based on the global scaling. The intramolecular interactions that lead to the slight compaction of the NAC region may result in self-shielding of this region. This could limit interactions with other α-synuclein monomers and potentially inhibit or slow down the formation of the amyloid fibrils found in Parkinson’s disease. Conversely, the terminal regions do not display such self-shielding, providing a large interaction surface for its many and diverse range of interaction partners.^53^ We found the smFRET data and the residue-scale CG-MD simulations to be in excellent agreement, allowing a molecular residue-scale view on the dominant interactions. The CG-MD simulations show that the intraregional compaction of the NAC region is the result of hydrophobic aliphatic contacts, mainly between valine and alanine residues. Interestingly, cation−π interactions between lysine and tyrosine contribute to the inter-regional C–N compaction. Only for one of the probed αS segments, we find a considerably larger compaction in smFRET experiments compared to the MD simulations. The observed expansion of the C-terminal region of the protein results from intraregional electrostatic repulsion. The presence of several prolines increases the chain stiffness, which also contributes to the expansion of the C-terminal region. Further, we foresee that fast CG-MD simulations alongside future smFRET experiments on IDPs can be used to identify regions in these proteins with higher propensities for secondary structure transitions. In this work, we have shown that CG-MD and smFRET measurements have an excellent agreement for disordered segments over a range of 10 to 133 residues in length. If a region exhibits a high probability of secondary structure formation, smFRET and CG-MD predictions will no longer be in agreement. This can be used to focus efforts of more computationally expensive all-atom MD simulations toward regions with the greatest likelihood of secondary structure transitions. Our study demonstrates the power of combining smFRET studies and MD simulations to unravel the key intra- and inter-regional interactions in highly dynamic IDPs at the amino acid level.

## Supporting Information Available

smFRET data of the 14 FRET-labeled αS constructs; *P*(*r*) and the FRET efficiency; and the intramolecular contact maps per residue type (PDF)
Movie depicting a full simulation (MP4)
