# Supplementary material for: Exploring Intra- and Inter-Regional Interactions in the IDP α-Synuclein Using smFRET and MD Simulations
Source: Biomacromolecules. 2023 Jul 5;24(8):3680–8. doi: 10.1021/acs.biomac.3c00404 (PMC10428166; doi:10.1021/acs.biomac.3c00404)
Supplement: Supplementary file 1 — bm3c00404_si_001.pdf [file bm3c00404_si_001.pdf]

Title: Exploring intra- and inter-regional interactions in the IDP α-synuclein using smFRET and MD simulations.

Authors: Gobert Heesink[1], Mirjam J. Marseille[1], Mohammad A.A. Fakhree[1], Mark D. Driver[2], Kirsten A. van Leijenhorst-Groener[1], Patrick R. Onck[2], Christian Blum[1], Mireille M.A.E. Claessens[1]

[1] Nanobiophysics, Faculty of Science and Technology, MESA + Institute for Nanotechnology and Technical Medical Centre, University of Twente, PO Box 217, 7500 AE Enschede, The Netherlands

[2] Micromechanics, Zernike Institute for Advanced Materials, University of Groningen, Groningen, The Netherlands

Supplementary information

smFRET data of the 14 FRET labelled αS constructs

$\alpha S_{9\text{-}18}$

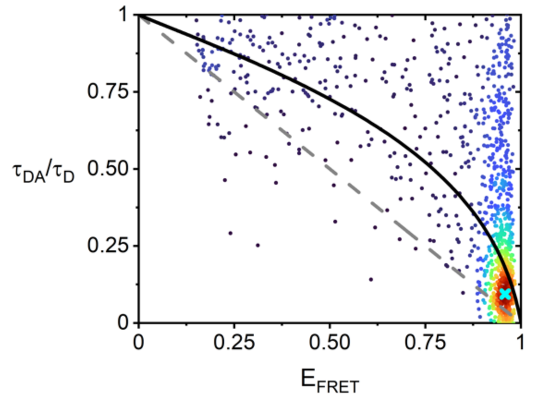

$\alpha S_{9\text{-}27}$

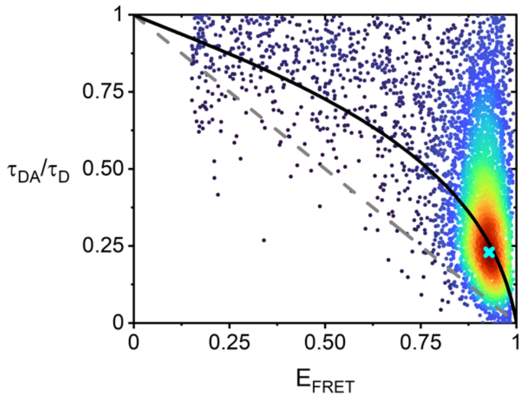

$\alpha S_{9\text{-}42}$

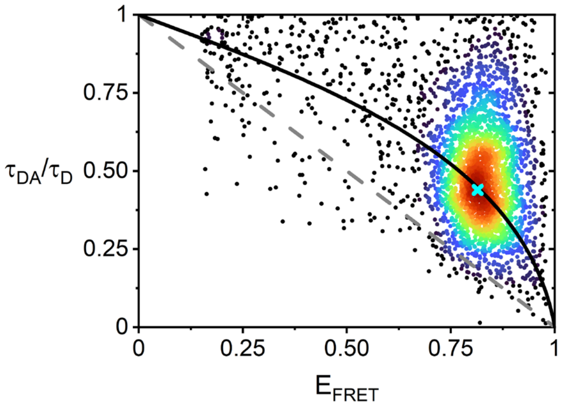

$\alpha S_{9\text{-}69}$

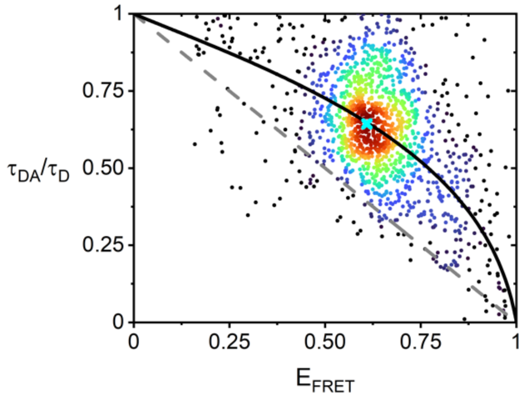

$\alpha S_{9\text{-}90}$

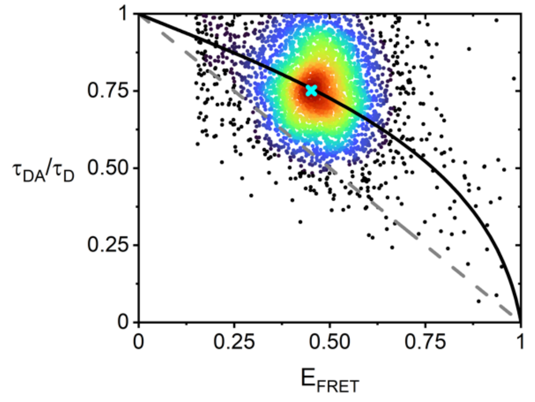

$\alpha S_{9\text{-}140}$

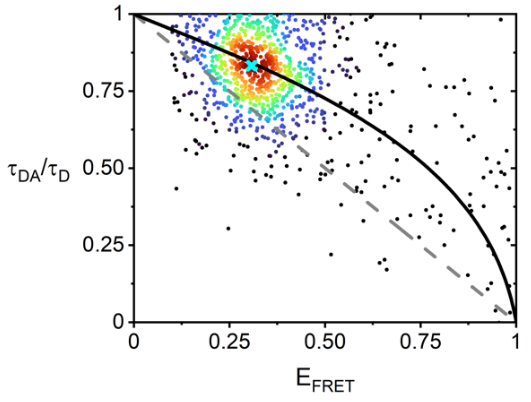

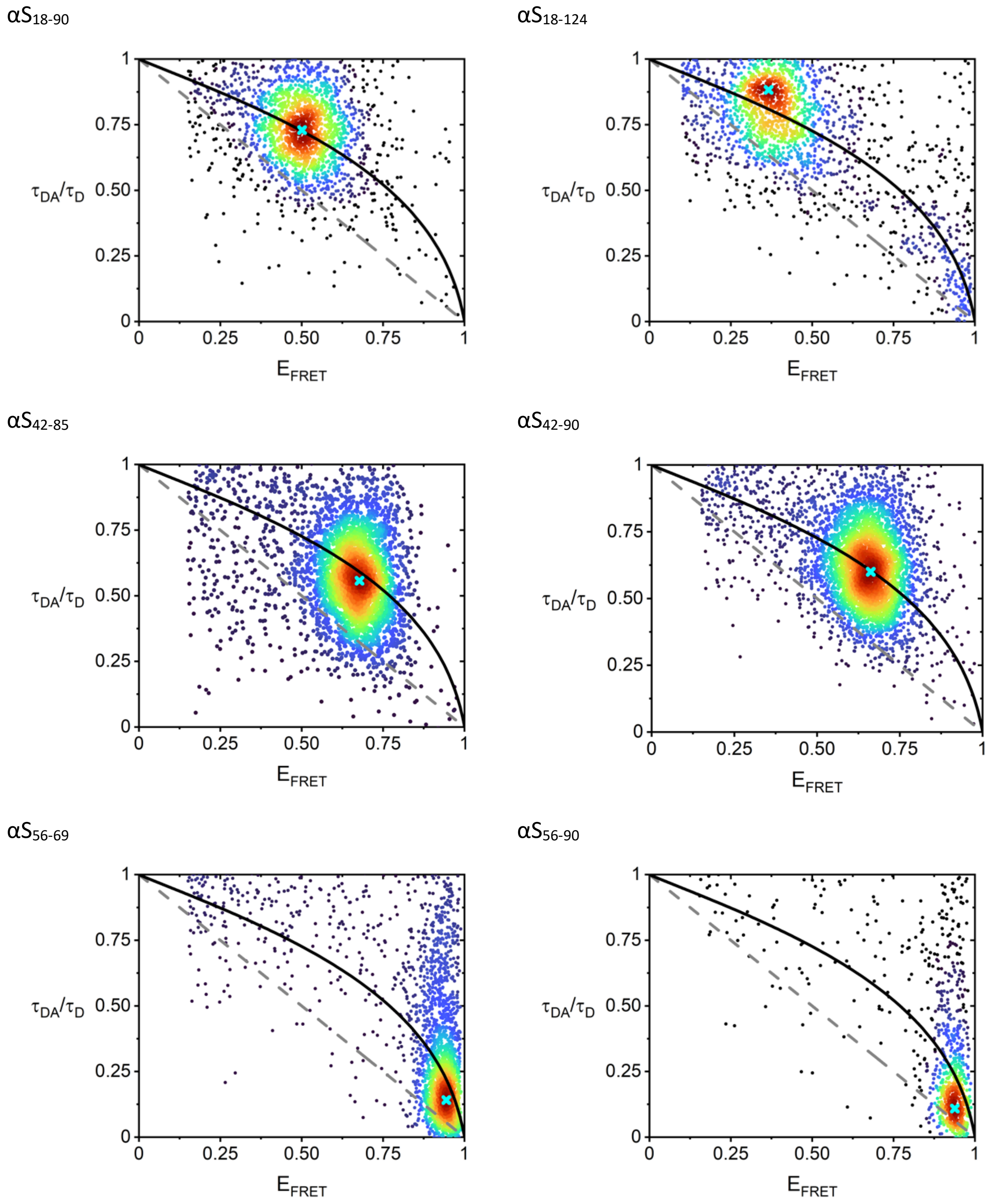

αS18-90
αS18-124
αS42-85
αS42-90
αS56-69
αS56-90
τDA/τD
EFRET
0
0.25
0.50
0.75
1

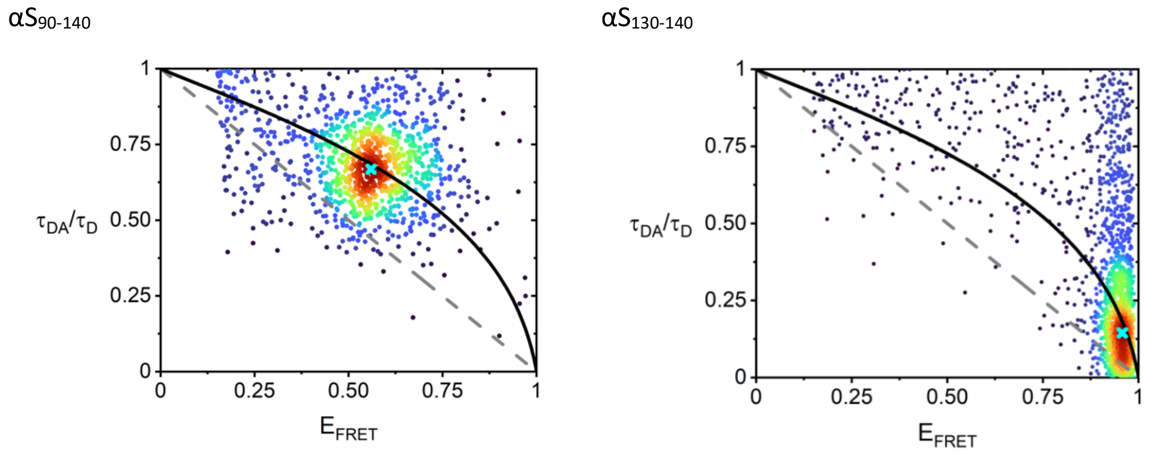

Figure S1. The smFRET burst clouds of the 14 αS constructs. The labeling positions are given above the figures. Each point in each plot represents one FRET burst. The colour scale from dark blue to dark red represents the density of data points from low to high density, respectively. The highest density centre of the data is indicated with a cyan cross. The dashed gray line represent the static line (absence of conformational dynamics). The black line represents the dynamic line and was calculated using the distance distribution expected for a self-avoiding polymer chain in a good solvent.

Normalization constants of $P(r)$

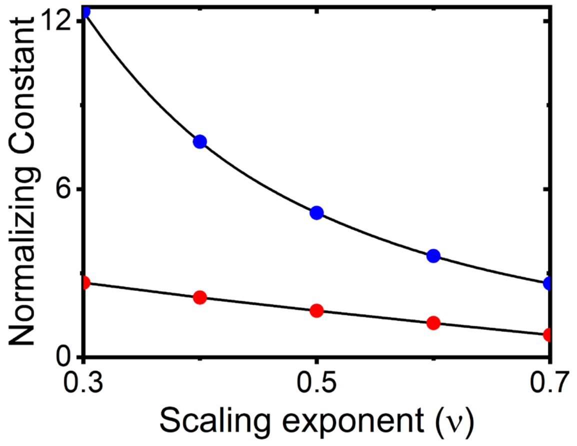

Figure S2. Calculated values of normalizing constants a and b in equation 2 as a function of v, for R = 5.5 nm. The blue and red dots represent individually calculated values for a and b, respectively. Their associated black lines are a spline and linear interpolation, respectively.

Visualization of $P(r)$ and $\int P(r) * E(r)dr$.

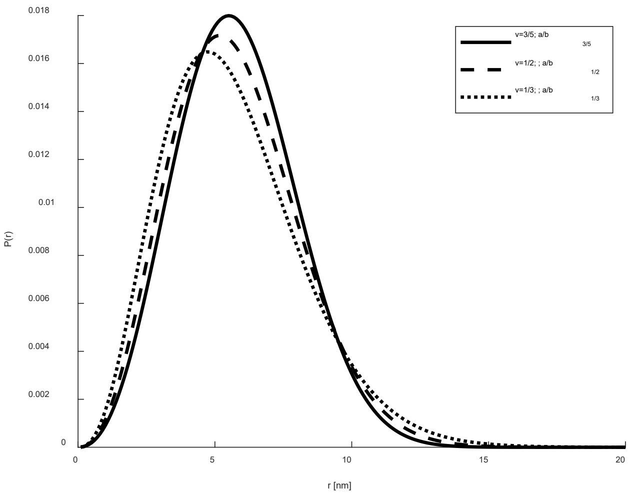

Figure S3a. Probability density function $P(r)$ according to equation 4 for R = 5.5 nm and v = 3/5, 1/2, and 1/3, given in solid, dashed and dotted lines, respectively.

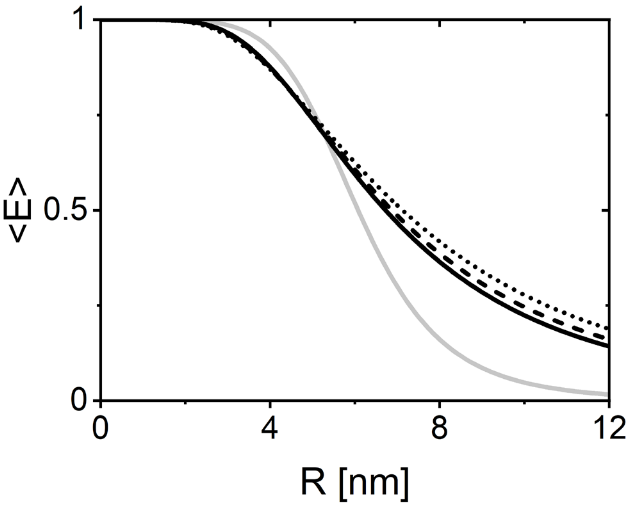

Figure S3b. Evolution of the FRET efficiency E_{FRET} with distance between the FRET donor and FRET acceptor fluorophore. *E*(*R*) depends on the distribution of distances between the FRET pairs.

Gray: no distribution, identical *R* for all FRET pairs in the ensemble.

Solid: distribution of distances *R* can be described as a polymer in good solvent (*v* = 3/5).

Dashed: distribution of distances *R* can be described as a polymer in ideal solvent (*v* = 1/2).

Dotted: distribution of distances R can be described as a polymer in bad solvent (*v* = 1/3).

Intramolecular contact maps

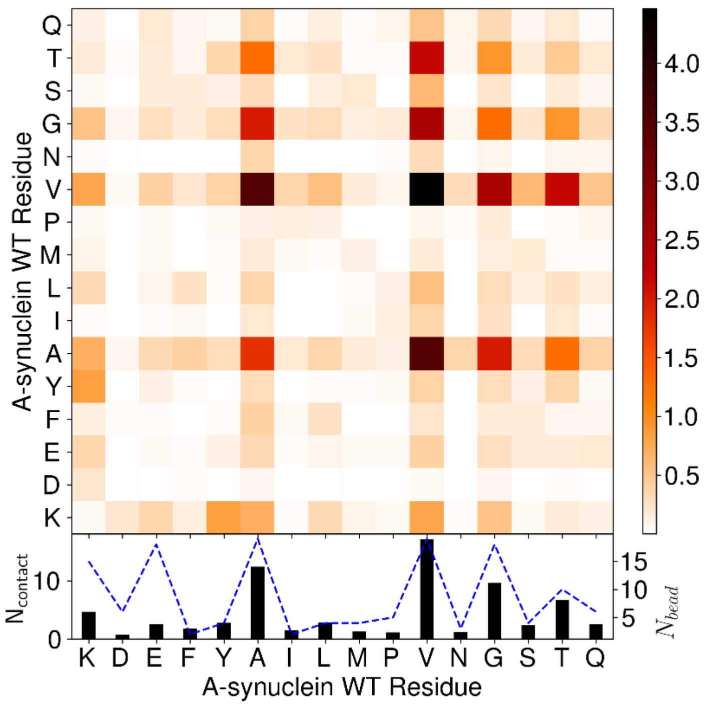

Figure S4a. Time averaged intramolecular contact map for αS by residue type. Amino acids with an abundance above 1% are displayed. The residue abundance is displayed by the blue line. Significant interactions between the alanine and valine residues are clearly visible in the contact map. Glycine and glutamic acid have comparable abundances to valine and alanine but reduced interactions.

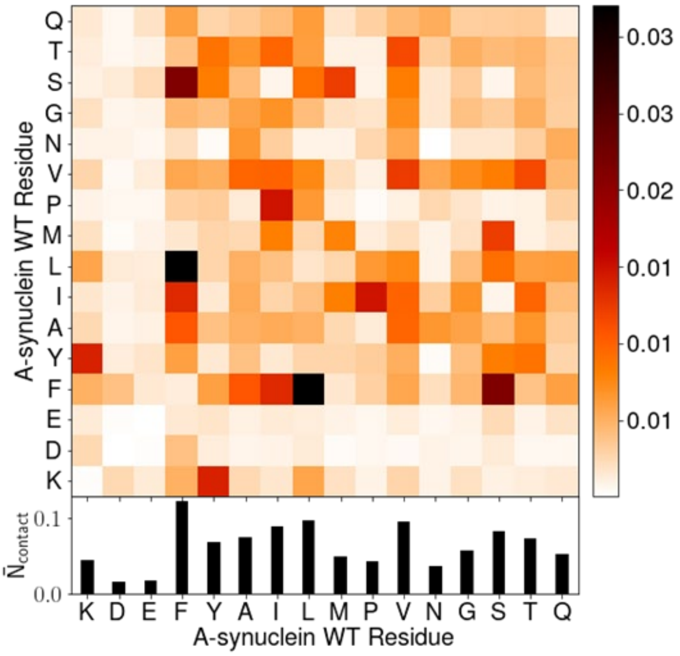

Figure S4b. Time and residue averaged intramolecular contact map for αS by residue type. Compared to Fig. 3a, the data has been normalised by the residue abundance. Residues with an abundance above 1% are displayed. The normalisation shows that valine provides a large number of contacts. Phenylalanine and leucine, which have a low abundance in αS, show a strong propensity for interactions.
